# Environmentally friendly novel macromolecular leveller imidazole-pentaerythritol glycidyl ether with high electroplating performance

**DOI:** 10.1098/rsos.261181

**Published:** 2026-10-28

**Authors:** Shoulong Zhang, David W. Zhang

**Affiliations:** College of Integrated Circuits and Micro-Nano Electronics, Fudan University, Shanghai, People's Republic of China

**Keywords:** leveller, chemical synthesis, copper electroplating, microvia

## Abstract

In this study, a novel environmentally friendly leveller, imidazole-pentaerythritol glycidyl ether (IMDPGE), which is free of sulfur (S) and other pollutant elements, was developed by chemical synthesis for blind-hole filling in acidic copper electroplating. The chemical structure of IMDPGE was characterized using Fourier transform infrared spectroscopy and mass spectrometry. The molecular properties of IMDPGE were investigated through quantum chemical calculations and molecular dynamics simulations, and its levelling performance was predicted accordingly. The electrochemical characteristics of IMDPGE, as well as its interactions with the accelerator bis-(3-sulfopropyl) disulfide (SPS) and the suppressor propyleneoxide (PO)-ethylene oxide (EO)-propylene oxide (PO), named PEP (EPE), were evaluated using galvanostatic potential transient measurements, cyclic voltammetry and linear scan voltammetry. The results demonstrated that IMDPGE exhibited strong polarization capability, which persisted even in the absence of SPS. Additionally, a kinetically controlled competitive adsorption existed between IMDPGE and SPS, while a synergistic inhibitory effect was observed between IMDPGE and EPE. The blind-hole filling performance of IMDPGE was verified through electroplating experiments. For the optimized EPE-SPS-IMDPGE additive system, a filling rate of 90.7% and a surface copper thickness of 7.7 µm were achieved after electroplating at a current density of 2 A dm^–^² for 30 minutes.

## 1. Introduction

With the rapid advancement of the electronic information industry, electronic products are increasingly becoming miniaturized, lightweight and high-performing. In line with this trend, the manufacturing technology of printed circuit boards (PCBs)—a critical interconnection and support component of electronic devices—is also encountering unprecedented challenges [1–4]. Notably, the precise processing and high-quality filling of microvias and blind vias have emerged as core technical challenges in the PCB manufacturing process [5–8].

In the acid copper electroplating process for blind via filling, high-quality void-free filling realized by preferential copper deposition at via bottoms highly depends on functional additives in the plating solution [9–11]. Additives act as the core factor determining electroplating performance. As vital functional additives, levellers improve the surface flatness of coatings through a differentiated inhibition mechanism. They can selectively adsorb on high current density areas to inhibit excessive local copper deposition, and exert little effect on low current density regions. Accordingly, uniform copper deposition is achieved to form dense and smooth copper coatings. Such planarization performance is crucial to the manufacture of high-precision electronic components such as PCBs [12–15].

Traditional levellers are mainly divided into quaternary ammonium salts, aromatic aldehyde and ketone derivatives, and heterocyclic compounds. Most of them contain toxic components, including organic sulfides and heavy metal ions. These hazardous substances seriously threaten the health of operators and cause environmental pollution [16–20]. Sulfur-containing additives are easy to decompose during electroplating and release toxic gases such as hydrogen sulfide. In addition, residual sulfur contaminants in waste plating solutions greatly increase the difficulty and cost of wastewater treatment, which is inconsistent with the concepts of green chemistry and sustainable development.

As shown in figure 1, this experiment produced a new type of leveller, imidazole-pentaerythritol glycidyl ether (IMDPGE), through a straightforward chemical synthesis method. In particular, none of the precursors of the reaction contain sulfur (S) and other pollutant elements. The resulting product underwent various characterization tests, and its performance was evaluated using electrochemical analyses, quantum chemical calculations and electroplating experiments for blind via filling.

**Figure 1.**
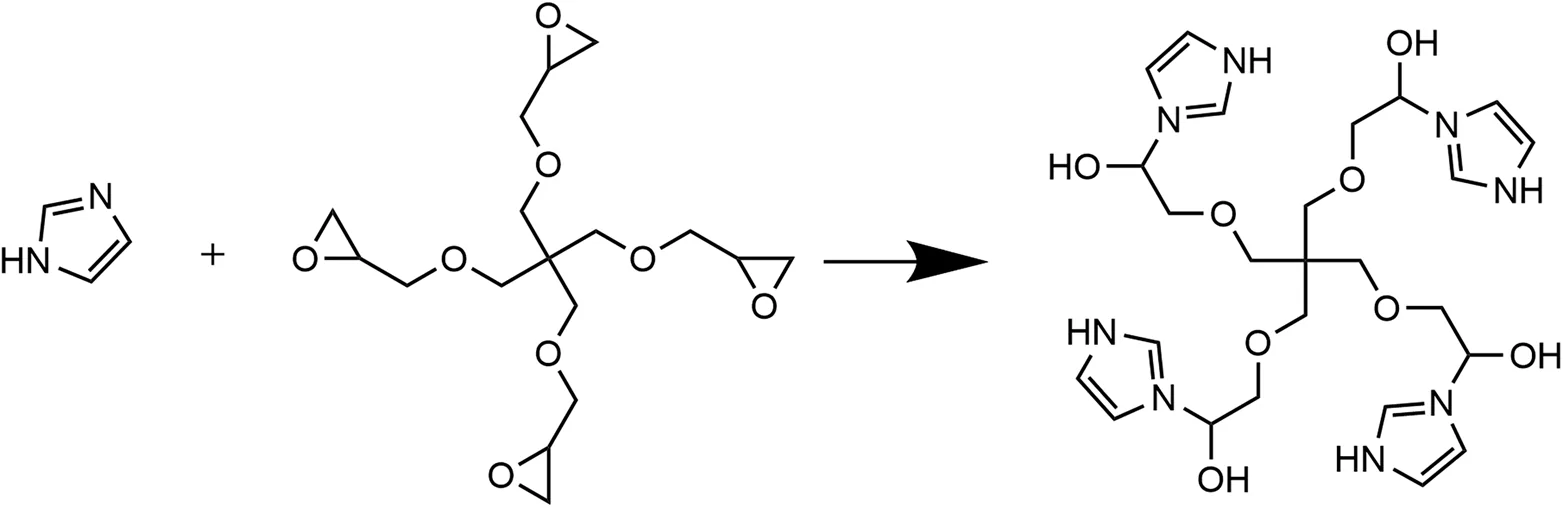
Molecular structure and synthetic route of IMDPGE.

## 2. Experiment

Electrochemical measurements in this experiment were conducted using a three-electrode system, comprising a copper electrode as the working electrode, a platinum electrode with an area of 7 mm² as the counter electrode and an Ag/AgCl electrode as the reference electrode. The virgin make-up solution (VMS) consisted of 200 g l^–1^ CuSO₄·5H₂O, 30 ml l^–1^ H₂SO₄ and 60 mg l^–1^ Cl^–^, and all experiments were carried out at 293 K.

### 2.1. Chemical synthesis characterization tools

Fourier transform infrared (FTIR) spectroscopy and mass spectrometry (MS) were employed as characterization techniques to confirm that the synthesized product is consistent with the target structure.

### 2.2. Quantum chemical calculations and molecular dynamics simulation

The molecular structure of IMDPGE was optimized using Gaussian 09W software with the B3LYP/6-31G (d,p) method. The optimized geometry and molecular orbitals were visualized using the Forcite module and Visual Molecular Dynamics (VMD) software. Adsorption of IMDPGE on the copper (111) surface was simulated at 293 K using Materials Studio software [21–24].

### 2.3. Electrochemical experiments

To evaluate the electroplating performance of the additive, galvanostatic potential transient measurements (GM) were used to measure the deposition potential at various rotating disc speeds. Before the tests, a pre-plating step was performed to deposit a thin copper layer on the platinum rotating disc electrode [25]. Cyclic voltammetry (CV) was conducted at a scan rate of 5 mV s^–1^, with the platinum rotating disc electrode (Pt-RDE) maintained at a constant rotation speed of 2000 r.p.m. [26]. Throughout the entire experiment, the electrolyte temperature was kept constant at 293 ± 1 K.

### 2.4. Copper electroplating

In a Haring cell with an effective volume of 1.5 l, a blind-hole PCB for electroplating was used as the cathode. The blind holes of the PCB had a diameter of 125 µm, a depth of 100 µm and a pre-plated copper layer thickness of 35 µm. The PCB was firmly fixed at the exact centre of the Haring cell using a dedicated fixture and connected to the negative terminal of the power supply. Meanwhile, two pieces of phosphorous copper anodes were fixed at both ends of the Haring cell and connected to the positive terminal of the power supply.

Prior to and throughout the entire electroplating process, bubbles were continuously introduced to both sides of the blind-hole board using an air pump. This approach aimed to promote forced convection of the electroplating solution, creating a significant difference in convection intensity between the inside and outside of the blind holes, thereby enhancing the filling efficiency of the electroplating solution within the holes. The electroplating experiment was conducted at current densities of 2 and 0.5 A dm^–2^, with plating durations of 30 and 120 minutes, respectively. The temperature during the electroplating process was maintained at 293 K.

After completing the electroplating process, the PCB subjected to anti-oxidation treatment was cut into metallographic sections. These sections were placed in a circular mould, and a uniformly mixed resin and curing agent were added in the correct proportions. The mould was then heated in an oven at 323 K for 2 hours. After cooling, the metallographic samples were obtained by demoulding.

## 3. Results and discussion

### 3.1. Chemical characterization results and toxicity analysis

Imidazole (IMD) and pentaerythritol glycidyl ether (PGE) were reacted in a three-necked flask at a molar ratio of 4 : 1. The reaction was conducted in a mixed solvent of water and ethanol, maintaining a temperature of 353 K for 12 hours.

The product was characterized by FTIR (figure 2) and MS. To identify the functional groups of the product, FTIR was performed over a wavenumber range of 400–4000 cm^–1^, involving 16 scan cycles in total. As shown in figure 2, the characteristic absorption peak of the epoxy group in IMDPGE at 913 cm^–1^ disappeared [27], indicating that the ring-opening reaction of PGE occurred. Additionally, a strong characteristic absorption peak of hydroxyl groups was observed in the wavenumber range of 3200–3600 cm^–1^, which further confirms the ring-opening of the epoxy group. The MS results showed that the weight-average molecular weight (Mw) of IMDPGE was 505 Daltons, and the polydispersity index (PDI, also referred to as the Mw distribution coefficient) was 1.338. The fact that the PDI value is close to 1 indicates that IMDPGE has high purity.

**Figure 2.**
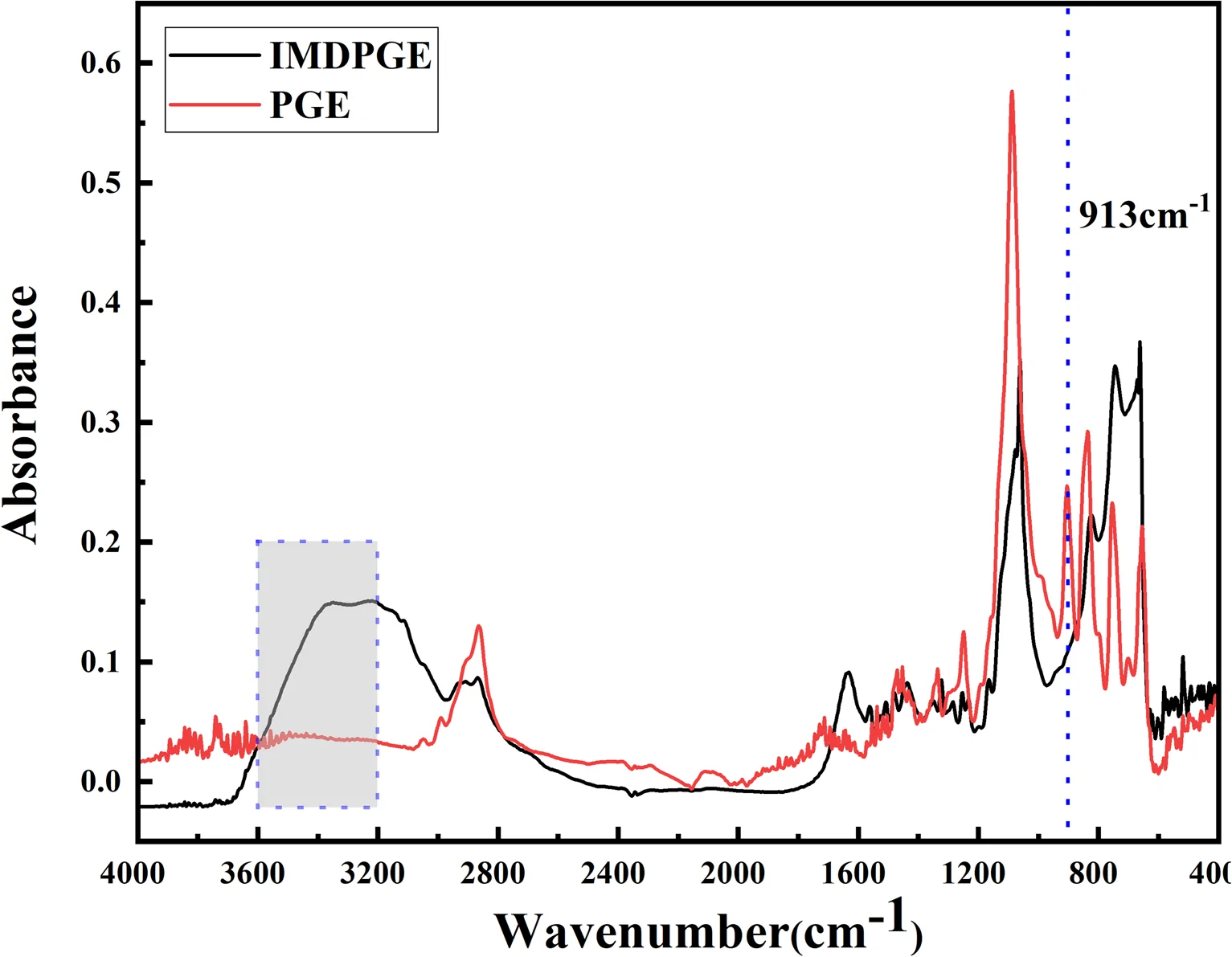
FTIR spectra of IMDPGE and PGE.

To evaluate the ecological effects of IMDPGE at its practical application concentration, and in accordance with the dosage used in subsequent electrochemical and electroplating experiments, this study adopted the 300-fold diluted sample as the test subject. *Lemna* sp. growth tests confirmed that this low-concentration IMDPGE was not only non-toxic but also exhibited a significant hormetic effect: after a 7-day exposure, the biomass of duckweed increased from an initial 0.013 to 0.067 g, representing a 186% increase compared with the blank control (0.0234 g). This suggests that residual organic compounds in the diluted IMDPGE can serve as usable carbon sources or nutrient factors, promoting biomass accumulation in aquatic plants. These results demonstrate that IMDPGE possesses excellent environmental compatibility at the recommended application concentration, aligning with the green chemistry principles of low toxicity, low residue and high environmental safety.

### 3.2. Quantum chemical calculations and molecular dynamics simulations

Figure 3a,b presents the distributions of the highest occupied molecular orbital (HOMO) and lowest unoccupied molecular orbital (LUMO) of IMDPGE obtained via quantum chemical calculations [28,29]. According to the frontier orbital theory, the electron transition behaviour between organic molecules and metals is closely related to the energy values of their HOMO (E_HOMO_) and LUMO (E_LUMO_). Generally, organic molecules with a higher E_HOMO_ value exhibit stronger electron-donating ability, while a lower E_LUMO_ value indicates more prominent electron-accepting properties [30,31]. Relevant studies have shown that the E_HOMO_ value largely determines the levelling effect of a leveller [32]. Through quantum chemical calculations, the E_HOMO_ value of IMDPGE was determined to be –7.500 eV, which is more favourable compared with that of Janus Green B (JGB) (–5.74 eV)—a widely used leveller in current applications. It can thus be inferred that IMDPGE may possess levelling performance superior to that of JGB [33]. Additionally, as observed in figure 3a, the HOMO of IMDPGE is mainly localized on the IMD ring. This distribution implies that the IMD ring can act as an active group that donates electrons to low-energy empty orbitals, thereby facilitating the formation of covalent bonds.

**Figure 3.**
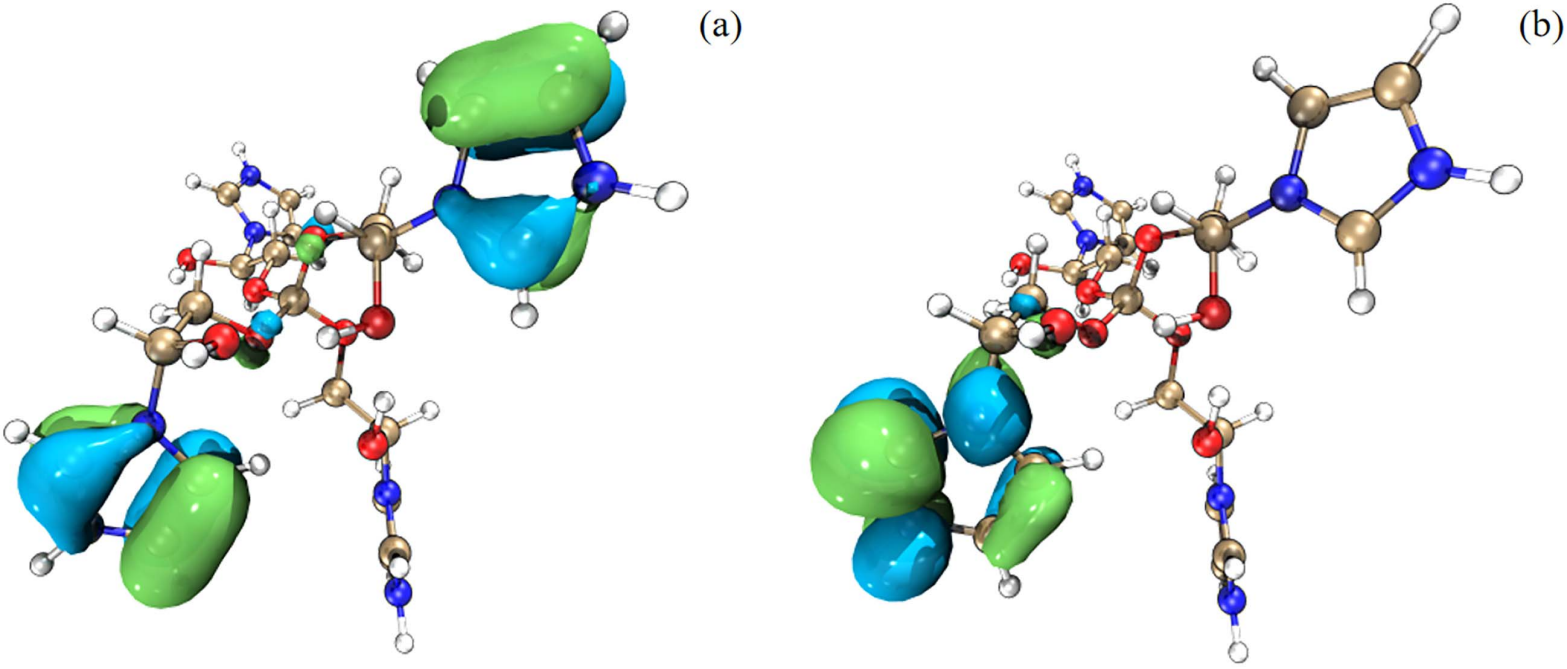
The frontier molecular orbitals: (a) HOMO and (b) LUMO of IMDPGE (gold: C, red: O, blue: N and white: H).

To investigate the adsorption behaviour of different levellers on the Cu (111) surface, molecular dynamics simulations were conducted in this study, with the results shown in figure 4. Figure 4a shows adsorption configuration of IMD on Cu (111), showing that IMD can adsorb onto the Cu (111) surface. Figure 4b shows adsorption configuration of IMDPGE, where both the IMD ring and oxygen atoms are tightly adsorbed onto the copper surface. Figure 4c shows adsorption configuration of JGB, revealing relatively loose adsorption of JGB molecules on the Cu (111) surface. The calculated adsorption energies for IMD, IMDPGE and JGB are –8.63, –104.94 and –95.93 kcal mol^–1^, respectively. These results demonstrate that IMDPGE exhibits higher adsorption activity towards inhibiting copper deposition compared with JGB. It can be observed that the IMD ring and oxygen atoms of IMDPGE are tightly adsorbed onto the Cu (111) surface. The Gaussian 09W and GaussView 5.0 software were employed to calculate the atomic charges of the IMDPGE molecule and generate its electrostatic potential (ESP) map, as presented in figure 5. For most levellers, their adsorption on copper surfaces typically relies on nitrogen (N) atoms as the active adsorption sites [34]. By contrast, IMDPGE uniquely contains oxygen (O) atoms, which can also serve as active adsorption sites, providing additional potential attachment points for the adsorption process.

**Figure 4.**
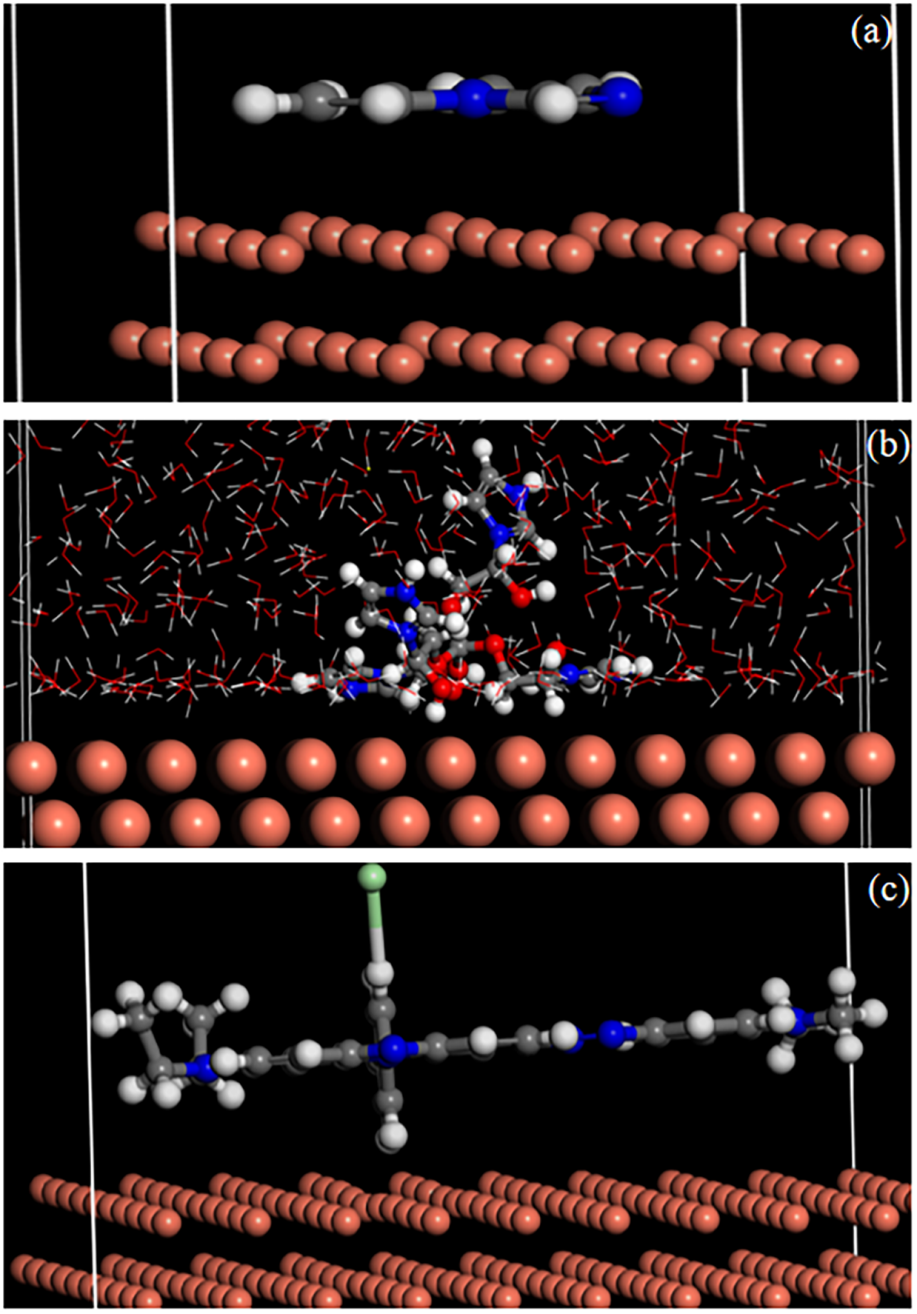
Adsorption configurations of (a) IMD, (b) IMDPGE and (c) JGB on the Cu (111) surface for molecular dynamics simulations (grey: C, red: O, blue: N, white: H and green: Cl).

**Figure 5.**
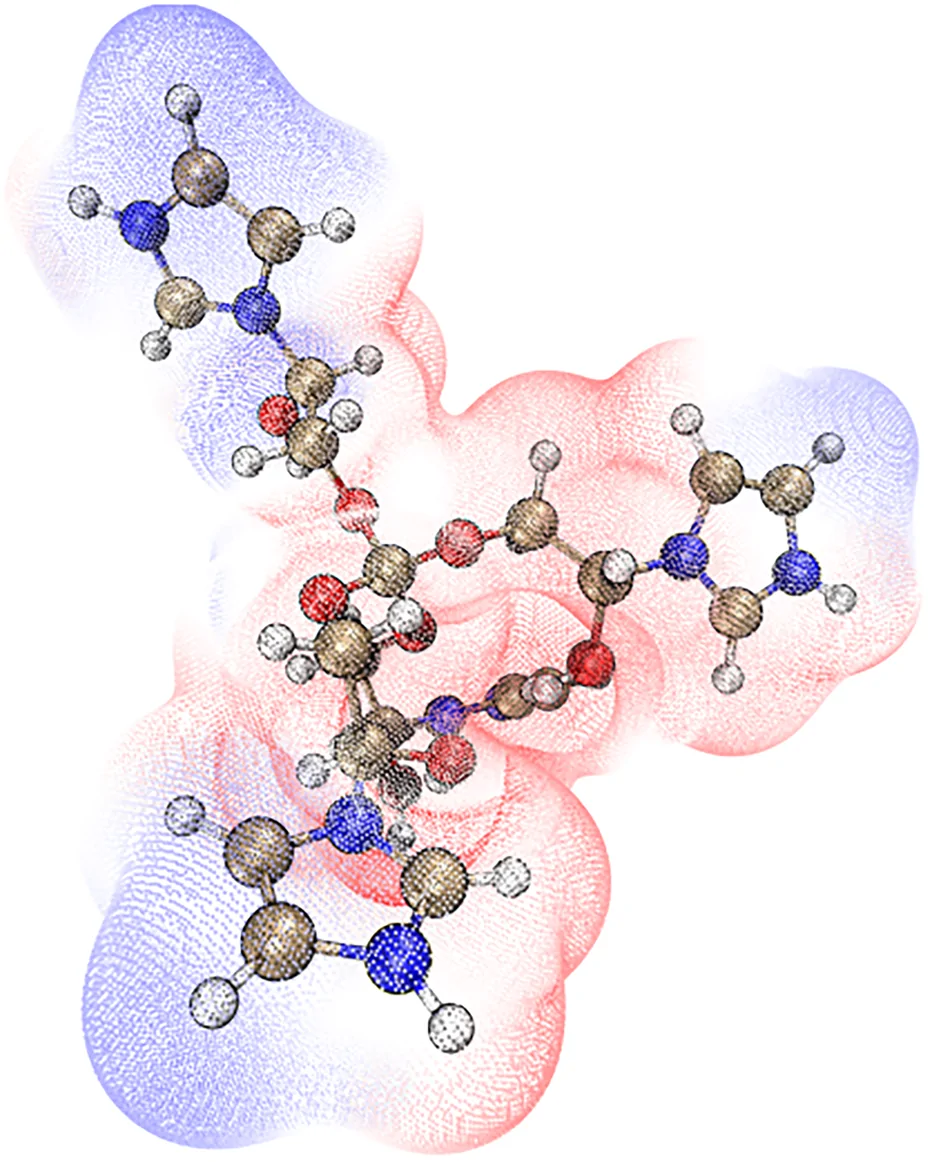
ESP-mapped molecular van der Waals (vdW) surface of IMDPGE (gold: C, red: O, blue: N and white: H).

Furthermore, IMDPGE has a relatively large molecular volume—a physical property that enables it to cover a broader area on the copper surface [35], effectively reducing the exposed area of copper and thereby significantly inhibiting the deposition of Cu^2+^ at the cathode. In addition, IMDPGE contains N^+^ species, which are adsorbed onto the cathode surface through electrostatic forces. It is important to emphasize that this electrostatic adsorption force is stronger than the physical adsorption force exerted by N atoms. It is the synergistic effect of the aforementioned factors that endows IMDPGE with high adsorption energy, making it a promising candidate for application as a high-efficiency leveller.

### 3.3. Electrochemical evaluation

The synthesized IMDPGE was added to VMS containing propyleneoxide (PO)-ethylene oxide (EO)-propylene oxide (PO), named PEP (EPE) and bis-(3-sulfopropyl) disulfide (SPS), relevant CV curves are presented in figure 6a. With the increase in IMDPGE concentration, the anodic current peak first decreased and then increased. When the VMS contained only EPE and SPS, the anodic current peak was 2.18 mA; when the concentration of added IMDPGE reached 20 mg l^–1^, the anodic current peak dropped to a minimum value of 0.819 mA. This indicates that IMDPGE can inhibit copper deposition.

**Figure 6.**
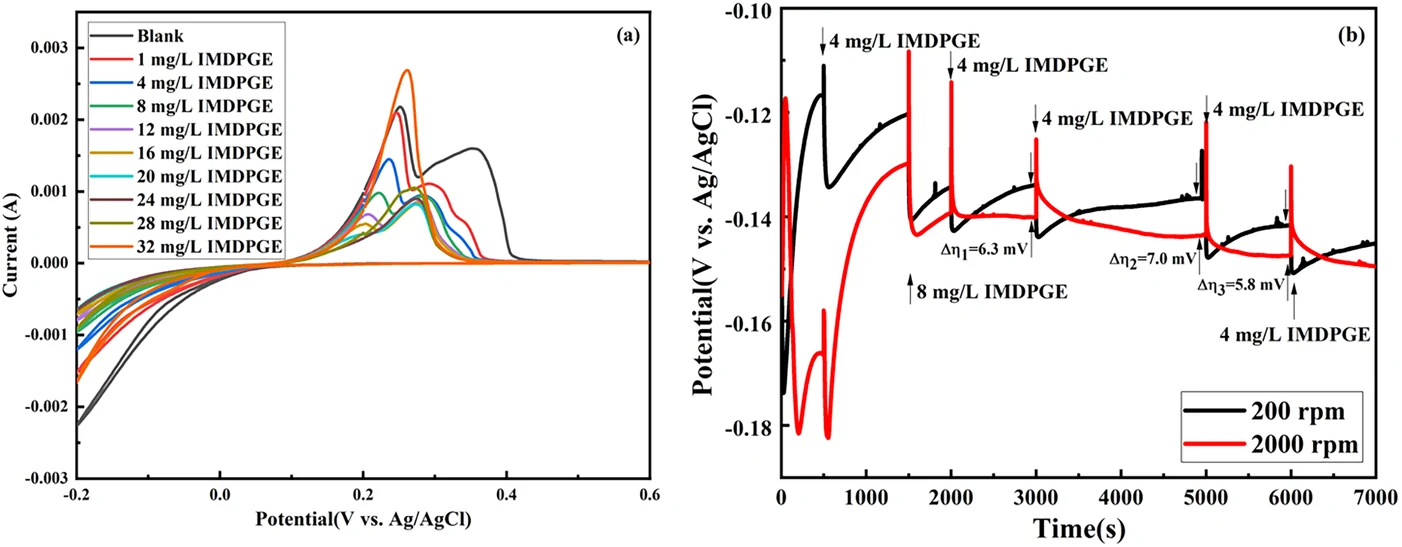
(a) CV and (b) GM curves obtained by adding IMDPGE with different concentrations to VMS containing EPE and SPS.

GM was conducted on the aforementioned plating bath system at different rotation speeds. As shown in figure 6b, the potential shifted negatively immediately after the addition of IMDPGE at 2000 r.p.m.; with the continuous addition of IMDPGE, a sustained negative shift in potential was observed. When the concentration of IMDPGE reached 24 mg l^–1^, further addition of IMDPGE no longer caused a significant potential drop, indicating that its inhibitory effect on copper deposition had reached a saturated state. At 200 r.p.m., the trend of electrode potential variation with IMDPGE concentration was essentially consistent with that at 2000 r.p.m. In copper electrodeposition, the indirect potential drop (Δη) is one of the key indicators for evaluating the performance of a leveller, defined as the difference between the absolute potential values of the leveller at high and low rotation speeds [36,37]. The maximum Δη value of IMDPGE was 7.0 mV. The results from CV and GM demonstrated that IMDPGE possesses the electrochemical properties required to be an effective leveller, and its optimal dosage was confirmed to be 20 mg l ^–1^.

Subsequently, GM was used to determine the optimal concentrations of EPE and SPS in the system.

Different concentrations of EPE were added to VMS containing 20 mg l^–1^ IMDPGE and 3 mg l^–1^ SPS. The obtained GM curves are shown in figure 7a. When 50 mg l^–1^ EPE was added, the cathodic potential showed a positive shift trend; this is because the concentration of EPE was too low, putting it at a disadvantage in competitive adsorption with SPS. As the concentration of EPE increased, the cathodic potential shifted negatively continuously, and the Δη value also increased gradually. When the EPE concentration was 300 mg l^–1^, the Δη reached a maximum value of 7.7 mV. Figure 7b presents the results obtained using the same experimental protocol to determine the optimal concentration of SPS. It can be observed that as the concentration of SPS increased, the electrode potential shifted positively continuously, which reflects the accelerating effect of SPS on the reduction of copper ions. The maximum Δη value was obtained when the SPS concentration was 4 mg l^–1^, reaching 7.5 mV.

**Figure 7.**
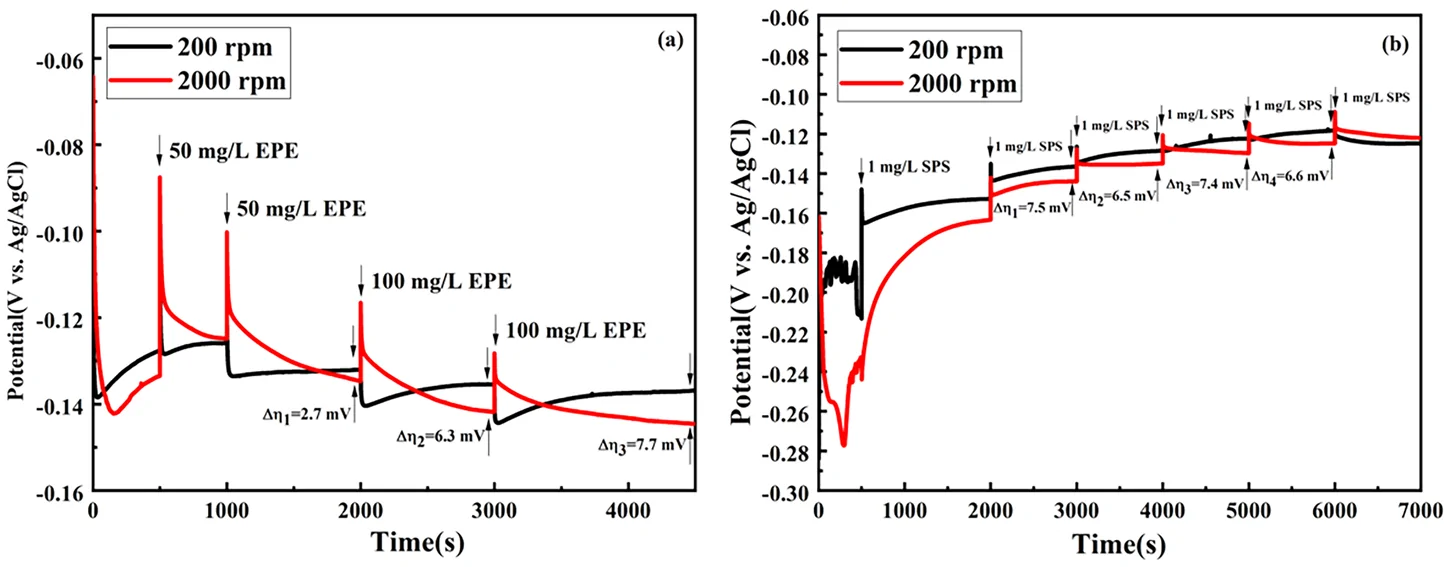
Determination of optimal concentrations of (a) EPE and (b) SPS by GM.

Based on the comprehensive analysis of the above electrochemical results, the optimal electroplating formulation was determined as follows: 20 mg l^–1^ IMDPGE + 300 mg l^–1^ EPE + 4 mg l^–1^ SPS.

The interaction between additives is a crucial part of studying the mechanism of additive action; rational use of such interactions is conducive to achieving super-filling of blind holes. Figure 8 shows the results of investigating the interaction between IMDPGE and other additives.

**Figure 8.**
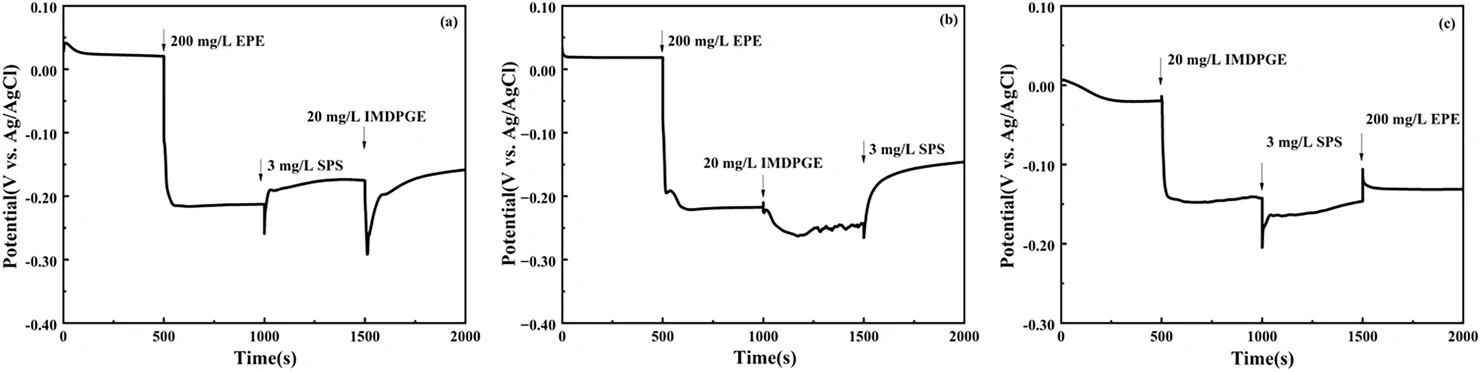
GMs with the successive addition of various additives at 2000 r.p.m.: (a) EPE + SPS + IMDPGE, (b) EPE + IMDPGE + SPS, and (c) IMDPGE + SPS + EPE.

In the 500–1500 s segment of figure 8a, the sequential addition of EPE and SPS caused polarization and depolarization phenomena, respectively. When IMDPGE was added to the VMS, the cathodic potential shifted negatively immediately. This is attributed to the fact that IMDPGE adsorbed onto the electrode surface promptly after being added to the VMS, thereby inhibiting copper deposition. However, in figure 8b, IMDPGE alone still induced a negative potential shift, indicating that IMDPGE can inhibit copper deposition even in the absence of SPS. This scenario is distinctly different from that of some traditional levellers (e.g. JGB), whose polarization effect can only be manifested in the presence of SPS [33,38]. The 1000–1500 s segment of figure 8b shows that SPS enhanced the inhibitory effect of IMDPGE, suggesting the existence of competitive adsorption between the two. As observed in the 500–1500 s segment of figure 8c, the addition of IMDPGE to the VMS containing EPE led to a further negative shift of the polarization potential, which demonstrates a synergistic inhibitory effect between EPE and IMDPGE. This result is consistent with the linear scan voltammetry (LSV) curve data in figure 9.

**Figure 9.**
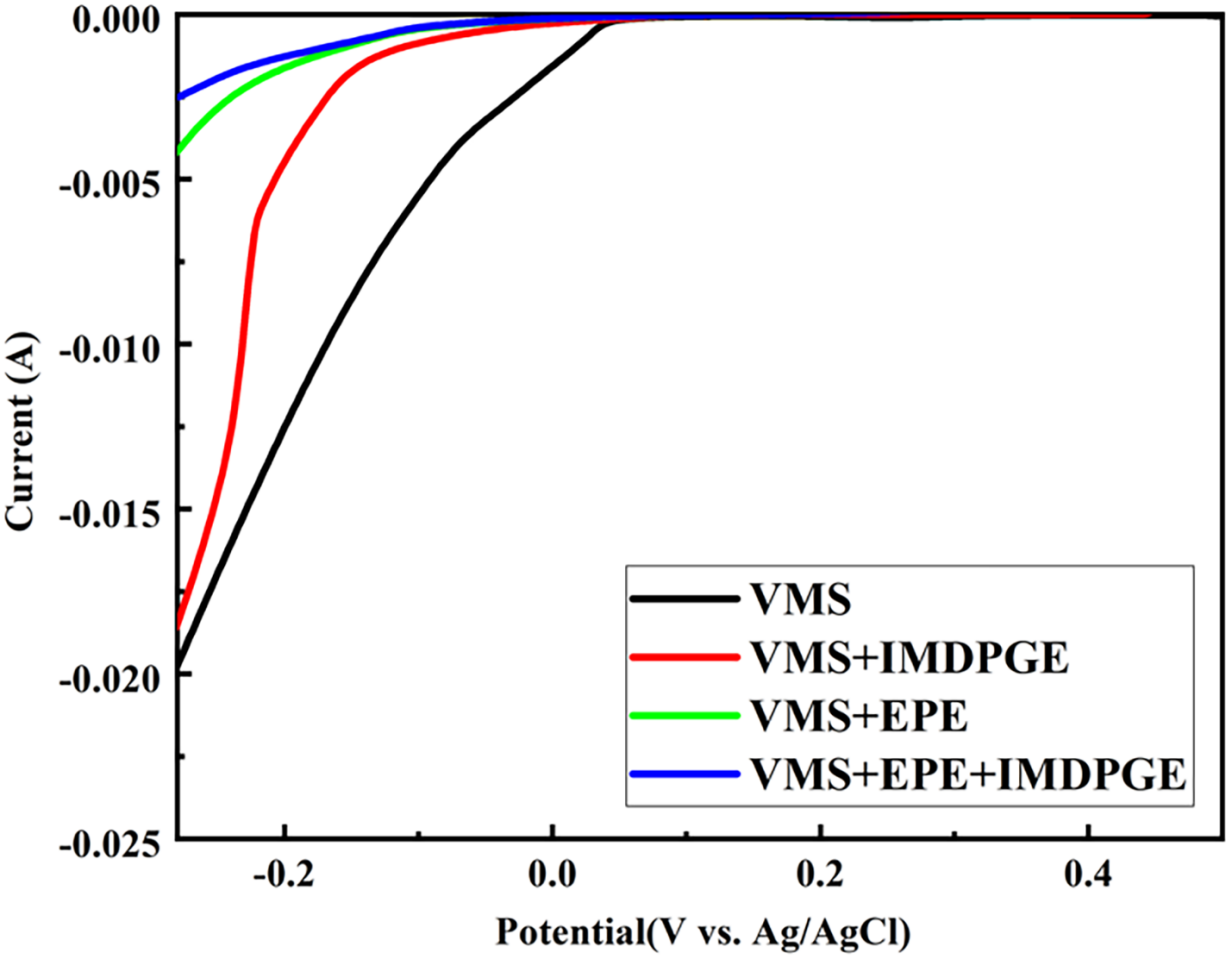
LSV curves obtained by adding EPE and IMDPGE in different orders.

In addition, combining the results from figure 8a–c, regardless of the addition order of the additives, the final stable potentials achieved were very similar, being −0.155, −0.149 and −0.144 V, respectively. This indicates that the additive system composed of SPS-EPE-IMDPGE is kinetically controlled during the electrodeposition process. Although competitive adsorption exists between SPS and IMDPGE during electroplating, a stable state can eventually be reached in all cases [39].

### 3.4. Electroplating experiments

Blind-hole PCBs pre-plated with a 35 µm seed layer were used as the cathode, placed at the centre of a Haring cell, and fixed with a holding device. The area of the PCB to be plated that was immersed in the electroplating solution was 60 × 100 mm^2^, and the PCB was connected to the negative terminal of a power supply. Phosphor-copper plates (anodes) were fixed at both ends of the cell, respectively, and connected to the positive terminal of the power supply.

Electroplating experiments were conducted at current densities of 2 and 0.5 A dm^–^²y. The composition of the electroplating solution was as follows: 60 mg l^–1^ Cl⁻, 300 mg l^–1^ EPE, 4 mg l^–1^ SPS and 20 mg l^–1^ IMDPGE. For further comparison, two sets of commercial copper electroplating additives were selected to perform blind-hole electroplating under the condition of 2 A dm^–^². After electroplating, the PCBs were subjected to cutting, sample preparation and grinding-polishing processes, then observed and analysed under a metallographic microscope. The results are shown in figure 10, and metallographic parameters are listed in table 1.

**Figure 10.**
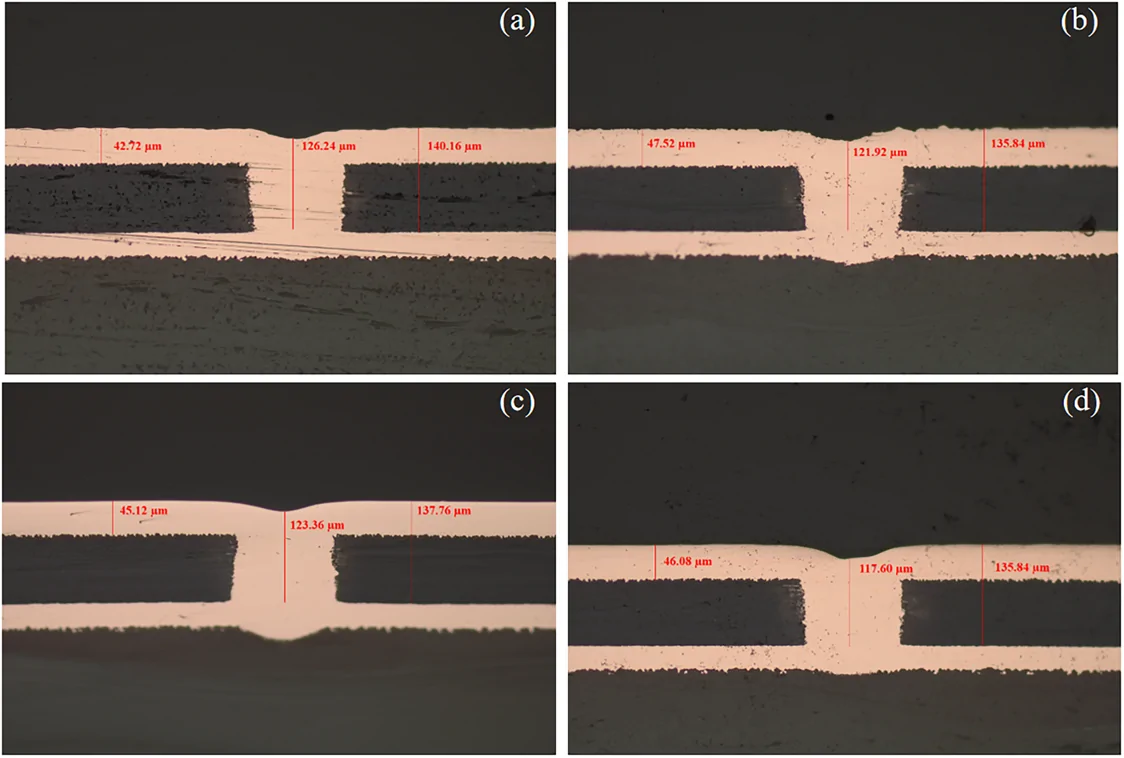
Cross-section images of microvias under different conditions: (a) IMDPGE, 2 A dm^–^², electroplating for 30 minutes; (b) IMDPGE, 0.5 A dm^–^², electroplating for 120 minutes; (c) L1, 2 A dm^–^², electroplating for 30 minutes; (d) L2, 2 A dm^–^², electroplating for 30 minutes.

**Table 1.** Plating via-filling rate and surface copper thickness under different conditions.

	IMDPGE, 2 A dm^–2^	IMDPGE, 0.5 A dm^–2^	L1, 2 A dm^–2^	L2, 2 A dm^–2^
filling rate (%)	90.7	89.7	89.5	86.6
surface copper thickness (µm)	7.7	12.5	10.1	9.0

As can be seen in figure 10a and table 1, at a current density of 2 A dm^–^², the electroplating filling rate of IMDPGE was 90.7%, and the surface copper thickness was 7.7 µm. By contrast, the filling rates and surface copper thicknesses of the commercial additives (shown in figure 10c and figure 10d) were 89.5% with 10.1 µm and 86.6% with 9.0 µm, respectively. It is evident that IMDPGE exhibits excellent electroplating performance and even outperforms commercial additives under specific conditions. By comparing figure 10a and figure 10b, it can be found that IMDPGE maintains a satisfactory hole-filling rate (89.7%) under different current densities; however, a lower current density leads to an increase in the surface copper thickness (12.5 µm).

To validate the universality of the optimized plating formulation under various electroplating conditions, supplementary experiments were conducted at 1 A dm^–^² for 60 minutes, 1.5 A dm^–^² for 40 minutes, 2.5 A dm^–^² for 24 minutes and 3 A dm^–^² for 20 minutes. The cross-sectional micrographs and the summarized data are presented in figure 11 and table 2, respectively. The results indicate that, under a constant charge basis, the plating formulation exhibits excellent blind via filling performance within a current density range of 1.5–2.5 A dm^–^², achieving a surface copper thickness below 9 µm and a filling rate exceeding 85%. Notably, the condition of 2 A dm^–^² for 30 minutes yields the optimal filling performance.

**Figure 11.**
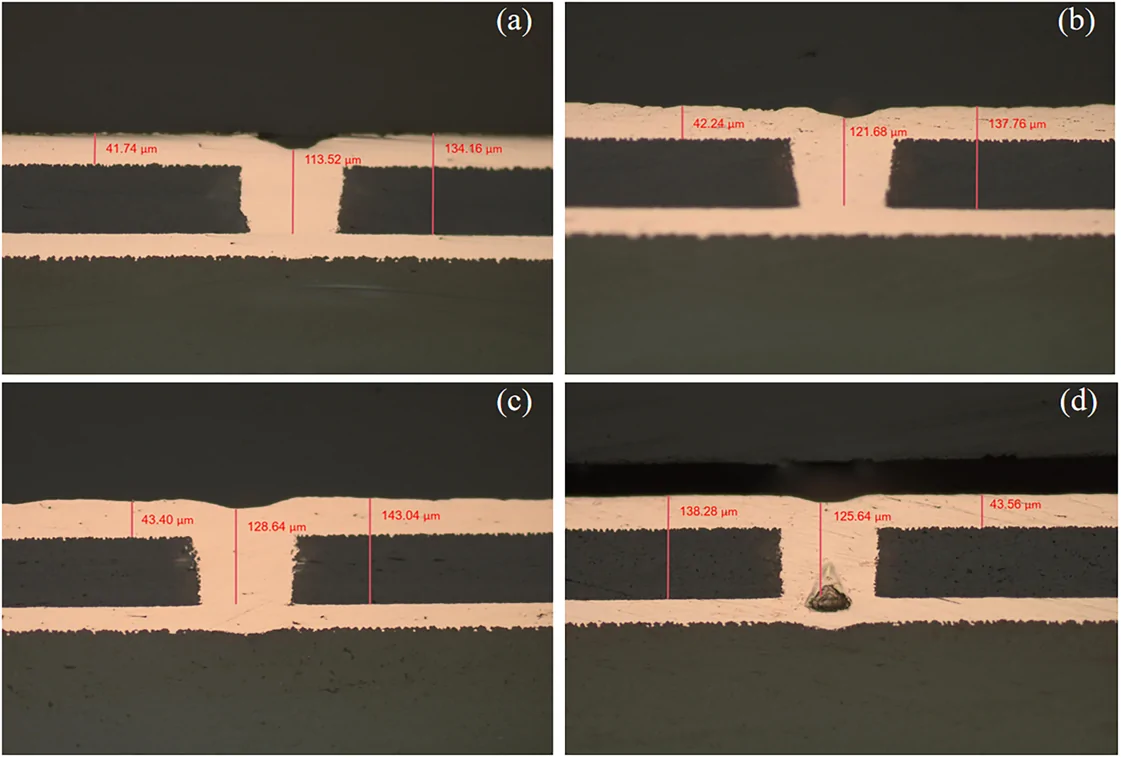
Cross-sectional micrographs of blind vias plated with IMDPGE as leveller under various electroplating conditions: (a) 1 A dm^–^², electroplating for 60 minutes; (b) 1.5 A dm^–^², electroplating for 40 minutes; (c) 2.5 A dm^–^², electroplating for 24 minutes; (d) 3 A dm^–^², electroplating for 20 minutes.

**Table 2.** Via-filling performance of IMDPGE under various electroplating conditions.

	1 A dm^–2^ × 60 minutes	1.5 A dm^–2^ × 40 minutes	2.5 A dm^–2^ × 24 minutes	3 A dm^–2^ × 20 minutes
filling rate (%)	90.7	89.7	89.5	86.6
surface copper thickness (µm)	7.7	12.5	10.1	9.0

Furthermore, to substantiate the reproducibility of our experimental data, multiple replicate experiments were performed at the optimal condition of 2 A dm^–^² for 30 minutes. The corresponding cross-sectional micrographs and via-filling parameters are provided in table S1 and figure S1 in the supplementary material. These replicate tests demonstrate that the optimized formulation exhibits remarkable stability and reproducibility under this specific condition, maintaining a surface copper thickness of 7–8 µm and a filling rate consistently greater than 90%.

To fully illustrate the exceptional capabilities of IMDPGE, a comparative experiment was performed at the optimal condition of 2 A dm^–^² for 30 minutes using IMDPGE and the commercial standard JGB as levellers. Metallographic analysis (figure 12) shows that JGB resulted in a much higher surface copper thickness (9.88 µm) and a lower filling rate (88%) compared with IMDPGE. The filling rate of IMDPGE is comparable to IMD-based leveller 1-(4-hydroxyphenyl)-2H-tetrazole-5-thione, which has already been reported [13]. To further evaluate the deposit quality, three-dimensional optical microscopy was employed. As presented in figure 13 and table 3, the arithmetic mean roughness (Sa) values roughness for the IMDPGE coating (0.315 µm) was substantially lower than that for JGB (0.556 µm), confirming that IMDPGE not only provides better filling but also yields a significantly smoother surface finish.

**Figure 12.**
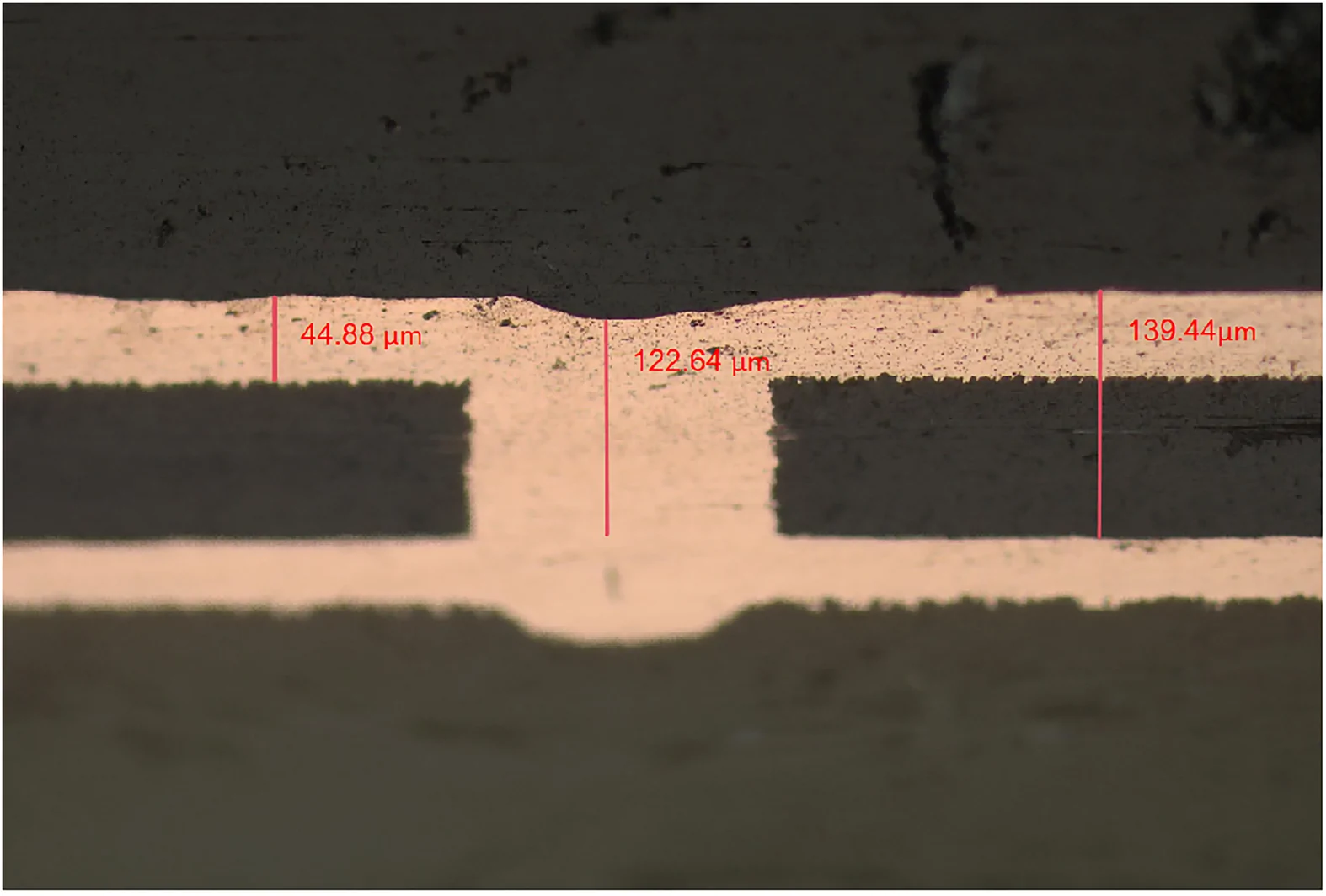
Cross-sectional micrograph of a blind via plated with JGB as leveller at 2 A dm^–^² for 30 minutes.

**Figure 13.**
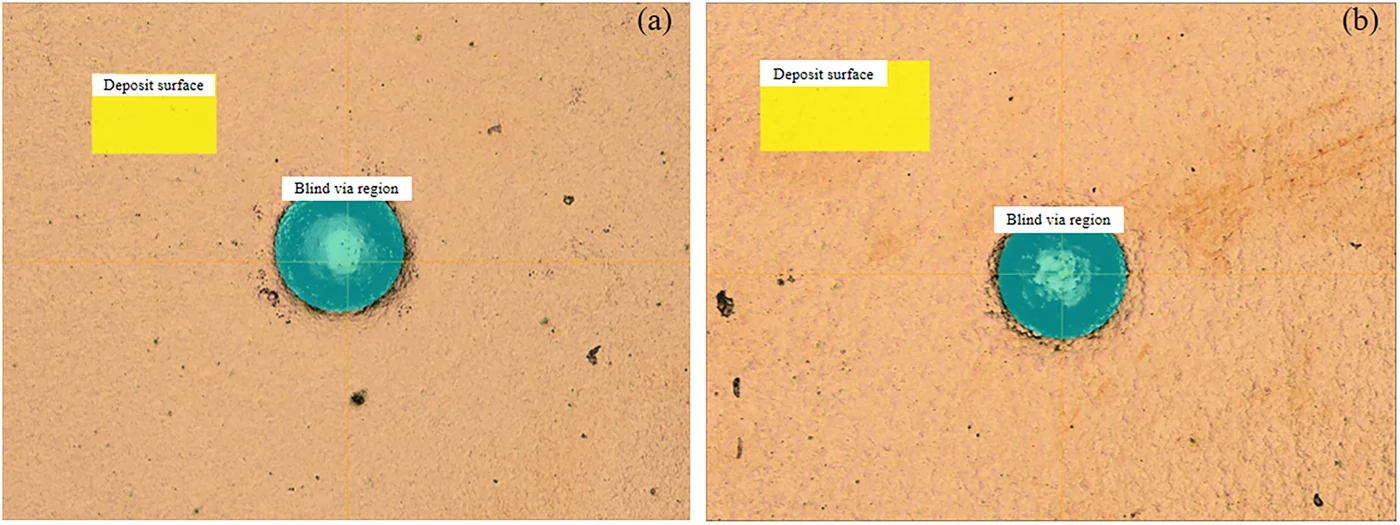
Three-dimensional surface topography of (a) IMDPGE and (b) JGB.

**Table 3.** Three-dimensional morphological parameters of copper deposits obtained with different levellers.

	IMDPGE	JGB
arithmetic mean roughness (Sa) (µm)	0.315	0.556
surface development ratio (Sdr) (%)	0.91	1.03

## 4. Conclusions and perspectives

In this experiment, an environmentally friendly leveller IMDPGE was obtained via chemical synthesis. Quantum chemical calculations revealed that the E_HOMO_ of IMDPGE was –7.50 eV, and the HOMO of IMDPGE was primarily localized on the IMD ring—enabling electron donation and covalent bond formation. Electrochemical experiments led to the determination of the optimal electroplating formulation using IMDPGE as the leveller: 300 mg l^–1^ EPE + 4 mg l^–1^ SPS + 20 mg l^–1^ IMDPGE. Further electrochemical studies indicated that IMDPGE and the inhibitor EPE exhibited a synergistic inhibitory effect. A maximum filling rate of 90.7% was achieved under the conditions of a current density of 2 A dm^–^² and an electroplating duration of 30 minutes. Additionally, the surface copper thickness was 7.7 µm, which was better than that of JGB.

In conclusion, the novel macromolecular leveller IMDPGE exhibits excellent blind-hole filling performance and levelling effect. Furthermore, the research on IMDPGE also provides a new direction for the exploration of environmentally friendly leveller. Building on these findings, our future work will focus on two key directions: (i) conducting in-depth investigations into the adsorption mechanisms of synthetic macromolecular levellers and establishing corresponding adsorption models, and (ii) extending the application of these macromolecular levellers to study their filling performance in through-holes and blind vias with smaller geometries.

## Supplementary Material



## Data Availability

All supporting raw electroplating and metallographic experimental data generated in this study are deposited in Zenodo [40]. No manuscript figures or tables were deposited in this repository, only original experimental raw data. Supplementary material is available online [41].

## References

[1] Srivastava V , Dubey S, Vaish R, Rajpurohit BS. 2025 Optimized fabrication of flexible paper-based PCBs with pencil and copper electroplating. Adv. Mater. Technol. 10, 2400688. (doi:10.1002/admt.202400688)

[2] Hou L , Zhao H, Lu Y. 2017 Fabrication of user-defined copper conductive patterns onto paper substrate for flexible electronics by combining wax patterning with electroless plating. J. Mater. Sci. Mater. Electron. 28, 4219–4228. (doi:10.1007/s10854-016-6044-6)

[3] Liao C , Zhang S, Chen S, Qiang Y, Liu G, Tang M, Tan B, Fu D, Xu Y. 2018 The effect of tricyclazole as a novel leveler for filling electroplated copper microvias. J. Electroanal. Chem. 827, 151–159. (doi:10.1016/j.jelechem.2018.08.042)

[4] Kumari V , Chandrakar M, Majumder MK. 2023 Performance analysis of cylindrical through silicon via with interfacial crack. In 24th Int. Symp. on Quality Electronic Design. New York, NY: IEEE. (doi:10.1109/ISQED57927.2023.10129400)

[5] Monier-Vinard E , Bissuel V, Daniel O, Brizoux M, Grivon A, Pires F. 2010 Impact of PCB via and micro-via structures on component thermal performances. In 16th Int. Workshop on Thermal Investigation of ICs and Systems (THERMINIC). New York, NY: IEEE.

[6] Liu F , Lu J, Sundaram V, Sutter D, White G, Baldwin DF. 2002 Reliability assessment of microvias in HDI printed circuit boards. IEEE Trans. Compon. Packag. Technol. 25, 254–259. (doi:10.1109/TCAPT.2002.1010014)

[7] Abbas SM , Yu Q, Pecht M. 2023 Influence of substrate on microvia structures in printed circuit boards during reflow. IEEE Access 11, 142 487–142 494. (doi:10.1109/ACCESS.2023.3342906)

[8] Heltzel S , Cauwe M, Bennett J, Rohr T. 2023 Advanced PCB technologies for space and their assessment using up-to-date standards. CEAS Space J. 15, 89–100. (doi:10.1007/s12567-021-00404-1)

[9] Wang Y-H , Senthil Raja D, Tsai D-H. 2024 Quantifying thiolated chemical additives for copper electroplating process. Anal. Chim. Acta 1307, 342608. (doi:10.1016/j.aca.2024.342608) 38719411

[10] Tao Z , Long Z, Tengxu L, Liu G, Tao X. 2022 The synergistic effects of additives on the micro vias copper filling. J. Electroanal. Chem. 918, 116456. (doi:10.1016/j.jelechem.2022.116456)

[11] Lin Z , Tao Z, Huang Y, Zhang J. 2025 Tween compounds as eco-friendly suppressors for acid copper electroplating applications of microvia. Electrocatalysis 16, 828–843. (doi:10.1007/s12678-025-00960-8)

[12] Xiao L *et al.* 2024 Investigation of azathioprine and its local-structure molecules as levelers for through-holes copper electroplating. Surf. Interfaces 53, 105073. (doi:10.1016/j.surfin.2024.105073)

[13] Teng X , Tao Z, Long Z, Liu G, Tao X. 2022 1-(4-hydroxyphenyl)-2*H*-tetrazole-5-thione as a leveler for acid copper electroplating of microvia. RSC Adv. 12, 16 153–16 164. (doi:10.1039/D2RA02274E) PMC9152711 35733656

[14] Lin Z , Tao X, Tengxu L, Tao Z. 2024 Evaluating copper plating process for filling micro vias with thin surface Cu by the 1-(4-carboxyphenyl)-5-mercapto-1H-tetrazole additive. J. Electroanal. Chem. 975, 118696. (doi:10.1016/j.jelechem.2024.118696)

[15] Huynh TMT *et al.* 2013 On the role of halides and thiols in additive-assisted copper electroplating. Electrochim. Acta 89, 537–548. (doi:10.1016/j.electacta.2012.10.152)

[16] Dow W-P , Li C-C, Su Y-C, Shen S-P, Huang C-C, Lee C, Hsu B, Hsu S. 2009 Microvia filling by copper electroplating using diazine black as a leveler. Electrochim. Acta 54, 5894–5901. (doi:10.1016/j.electacta.2009.05.053)

[17] Huang Q , Baker-O'Neal BC, Parks C, Hopstaken M, Fluegel A, Emnet C, Arnold M, Mayer D. 2012 Leveler effect and oscillatory behavior during copper electroplating. J. Electrochem. Soc. 159, D526–D531. (doi:10.1149/2.020209jes)

[18] Meng Y , Zhou M, Huang W, Min Y, Shen X, Xu Q. 2022 Benzyl-containing quaternary ammonium salt as a new leveler for microvia copper electroplating. Electrochim. Acta 429, 141013. (doi:10.1016/j.electacta.2022.141013)

[19] Zhu Q-S , Ding Z-F, Wei X-F, Guo J-D, Wang X-J. 2023 Effect of leveler on performance and reliability of copper pillar bumps in wafer electroplating under large current density. Microelectron. Reliab. 146, 115030. (doi:10.1016/j.microrel.2023.115030)

[20] Jin L , Song T, Shi Z-Y, Bao R-A, Yang F-Z. 2025 Study on the structural properties of an imidazole derivative synthesized based on the Schiff base reaction and its effects as a leveler on microvia copper electroplating. J. Electroanal. Chem. 998, 119543. (doi:10.1016/j.jelechem.2025.119543)

[21] Gao W , Gao B, Zhao Y, Lu Y, Chen R, An M, Liu A. 2025 Theoretical studies of the additives for copper electroplating in PCB by DFT and MD simulations. New J. Chem. 50, 256–264. (doi:10.1039/d5nj02183a)

[22] Yuan B , Zhou W, Li X, Xie Y, Yin X, Chen X, Shen D, Wang L. 2023 Investigation of synthesized carbazole derivative Cz-BPDB as a high-performance leveler for copper electroplating. Surf. Coat. Technol. 463, 129526. (doi:10.1016/j.surfcoat.2023.129526)

[23] Yuan B , Chen X, Zhao Y, Zhou W, Li X, Wang L. 2023 Unveiling the potential and mechanisms of 3, 3′-bicarbazole-based quaternary ammonium salts as levelers. Electrochim. Acta 471, 143345. (doi:10.1016/j.electacta.2023.143345)

[24] Ishwarya S , Nagaswarupa HP, Naik YV, Naik R, Koyyada G, Kim JH, Pandiaraj S, Alzahrani KE. 2026 DFT based design and one-pot synthesis of Ca_2_V_2_O_7_/ZnO nanocomposite for multifunctional supercapacitor and photocatalysis applications. Mater. Chem. Phys. 349, 131778. (doi:10.1016/j.matchemphys.2025.131778)

[25] Vijaykumar CJ , Bulla SS, Chavan C, Nagaswarupa HP, Bhajantri RF, Pushpa B, Naik R. 2026 Comprehensive review of solid-state batteries: theoretical conceptualization to sustainable energy storage technologies. J. Energy Storage 151, 120431. (doi:10.1016/j.est.2026.120431)

[26] Prakash G *et al.* 2026 High-performance solution-combustion synthesized V_2_O_5_/NiO nanocomposites for supercapacitor applications: comparative electrochemical evaluation. J. Mater. Chem. C 14, 8238–8255. (doi:10.1039/d5tc03543k)

[27] Zhang J , Yang H, Shen G, Cheng P, Zhang J, Guo S. 2010 Reduction of graphene oxide via L-ascorbic acid. Chem. Commun. 46, 1112–1114. (doi:10.1039/B917705A) 20126730

[28] Barrahi A *et al.* 2026 Adsorption and inhibition mechanism of pyrazole derivatives on carbon steel: combined electrochemical, surface, and DFT/MD study. RSC Adv. 16, 15 531–15 552. (doi:10.1039/D6RA00508J) PMC13000905 41868347

[29] Lai Z *et al.* 2018 Computational analysis and experimental evidence of two typical levelers for acid copper electroplating. Electrochim. Acta 273, 318–326. (doi:10.1016/j.electacta.2018.04.062)

[30] Feng L , Yang H, Wang F. 2011 Experimental and theoretical studies for corrosion inhibition of carbon steel by imidazoline derivative in 5% NaCl saturated Ca(OH)_2_ solution. Electrochim. Acta 58, 427–436. (doi:10.1016/j.electacta.2011.09.063)

[31] Finšgar M , Lesar A, Kokalj A, Milošev I. 2008 A comparative electrochemical and quantum chemical calculation study of BTAH and BTAOH as copper corrosion inhibitors in near neutral chloride solution. Electrochim. Acta 53, 8287–8297. (doi:10.1016/j.electacta.2008.06.061)

[32] Wang C , Zhang J, Yang P, An M. 2013 Electrochemical behaviors of Janus Green B in through-hole copper electroplating: an insight by experiment and density functional theory calculation using Safranine T as a comparison. Electrochim. Acta 92, 356–364. (doi:10.1016/j.electacta.2013.01.064)

[33] Lai Z , Wang S, Wang C, Hong Y, Zhou G, Chen Y, He W, Peng Y, Xiao D. 2018 A comparison of typical additives for copper electroplating based on theoretical computation. Comput. Mater. Sci. 147, 95–102. (doi:10.1016/j.commatsci.2017.11.049)

[34] Wang C , An M, Yang P, Zhang J. 2012 Prediction of a new leveler (N-butyl-methyl piperidinium bromide) for through-hole electroplating using molecular dynamics simulations. Electrochem. Commun. 18, 104–107. (doi:10.1016/j.elecom.2012.02.028)

[35] Reid JD , Zhou J. 2007 Impact of leveler molecular weight and concentration on damascene copper electroplating. ECS Trans. 2, 77–92. (doi:10.1149/1.2408866)

[36] Dow W-P , Chen H-H, Yen M-Y, Chen W-H, Hsu K-H, Chuang P-Y, Ishizuka H, Sakagawa N, Kimizuka R. 2008 Through-hole filling by copper electroplating. J. Electrochem. Soc. 155, D750–D757. (doi:10.1149/1.2988134)

[37] Chiu Y-D , Dow W-P. 2013 Accelerator screening by cyclic voltammetry for microvia filling by copper electroplating. J. Electrochem. Soc. 160, D3021–D3027. (doi:10.1149/2.006312jes)

[38] Dow W-P , Li C-C, Lin M-W, Su G-W, Huang C-C. 2009 Copper fill of microvia using a thiol-modified Cu seed layer and various levelers. J. Electrochem. Soc. 156, D314–D320. (doi:10.1149/1.3147273)

[39] Vetter KJ . 2013 Electrochemical kinetics: theoretical aspects. New York, NY: Elsevier.

[40] Zhang S . 2026 Supporting raw data for manuscript: Environmentally friendly novel macromolecular leveler IMDPGE with high electroplating performance. Zenodo. (doi:10.5281/zenodo.20773700)

[41] Zhang S , Zhang DW. 2026 Supplementary material from: Environmentally friendly novel macromolecular leveller imidazole-pentaerythritol glycidyl ether with high electroplating performance. Figshare. (doi:10.6084/m9.figshare.c.8745906)

