# Supplementary material for: Environmentally friendly novel macromolecular leveller imidazole-pentaerythritol glycidyl ether with high electroplating performance
Source: R Soc Open Sci. 2026 Oct 28;13(10):261181. doi: 10.1098/rsos.261181 (PMC13714404; doi:10.1098/rsos.261181)
Supplement: Supplementary file 1 [file rsos.261181.zip › rsos.261181/rsos261181_si_001.pdf]

Table 1. Via-Filling Parameters of IMDPGE Obtained from Repeated Electroplating Tests at 2 A/dm^2

	Filling rate (%)	Surface Copper Thickness (μm)
(a)	90.7	7.72
(b)	92.7	7.48
(c)	94.2	7.82
(d)	94.1	7.48
(e)	92.4	7.50
(f)	91.6	7.92
(g)	91.8	7.48
(h)	92.7	7.24
(i)	90.5	7.78
Mean	92.3	7.60
SD	1.31	0.22

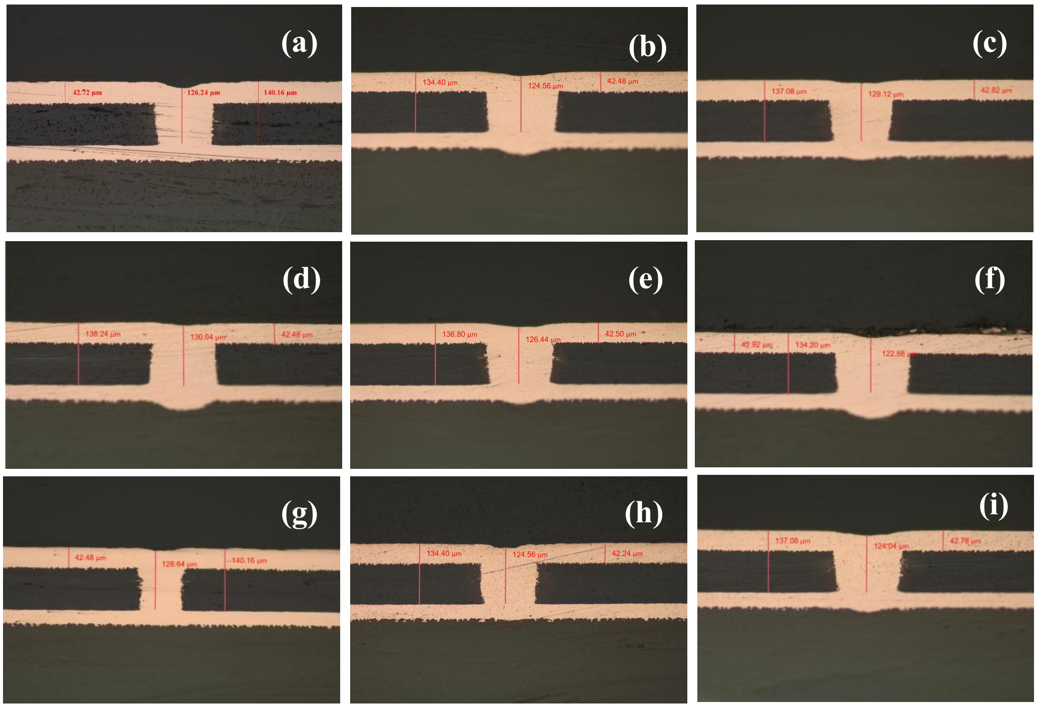

Figure 1. Metallographic Cross-Sections of IMDPGE Obtained from Repeated Electroplating Tests at 2 Adm^2
